# HOXC13-AS Induced Extracellular Matrix Loss *via* Targeting miR-497-5p/ADAMTS5 in Intervertebral Disc

**DOI:** 10.3389/fmolb.2021.643997

**Published:** 2021-07-02

**Authors:** Wanli Jing, Wei Liu

**Affiliations:** ^1^ Department of Orthopaedics, Tianjin First Central Hospital, Tianjin, China; ^2^ Department of Orthopaedics, Baodi Peopele’s Hospital, Tianjin, China

**Keywords:** HOXC13-AS, intervertebral disc, miR-497-5p, ADAMTS5, lncRNA

## Abstract

**Background/Aims:** LncRNAs are a new modulator in the development of intervertebral disc degeneration. However, the functional role and mechanism of HOXC13-AS in intervertebral disc degeneration remain unclear.

**Methods:** qRT-PCR analysis was performed to measure the relative expression levels of HOXC13-AS and miR-497-5p, and the levels of IL-1β, IL-6, and TNF-α in the medium supernatant were analyzed by ELISA. The related mechanism between HOXC13-AS and miR-497-5p was detected by luciferase assays.

**Results:** The results revealed that TNF-α and IL-1β induced HOXC13-AS expression in NP cells. HOXC13-AS was overexpressed in IDD specimens compared to control specimens, and higher expression of HOXC13-AS was correlated with high Pfirrmann scores. Ectopic expression of HOXC13-AS promoted MMP-3 and ADAMTS4 and inhibited aggrecan and collagen II expression in NP cells. Furthermore, overexpression of HOXC13-AS increased the expression of inflammatory cytokines, including IL-1β, IL-6, and TNF-α. Our results demonstrated that TNF-α and IL-1β induced ADAMTS5 expression and suppressed miR-497-5p expression. miR-497-5p was downregulated in IDD specimens compared to control specimens, and the lower expression of miR-497-5p was correlated with high Pfirrmann scores. The miR-497-5p level was negatively proportional to HOXC13-AS expression in IDD specimens. Luciferase analysis data indicated that ADAMTS5 was a direct target gene of miR-497-5p. HOXC13-AS induced inflammatory cytokine expression and ECM degradation by modulating miR-497-5p/ADAMTS5.

**Conclusion:** HOXC13-AS may be a treatment target for IDD.

## Introduction

Low back pain (LBP) is a common disorder that is experimentally and clinically concerning (Setton and Chen, 2004; Seguin et al., 2008; Inoue and Espinoza Orias, 2011). The etiology of LBP is still unclear, and the major cause of LBP is IDD (intervertebral disc degeneration) (Roughley, 2004; Raj, 2008; Loreto et al., 2011). IDD is usually considered a natural process in intervertebral disc aging, but several cases have indicated accelerated disc degeneration according to genetic and environmental factors (Johnson and Roberts, 2003; Le Maitre et al., 2005; Li et al., 2012). Disc cells secrete anomalous inflammatory cytokines due to smoking, excessive biomechanical loading, genetic predisposition, aging, and decreased nutrient transport, which can result in disc cell apoptosis, autophagy and senescence (Furukawa et al., 2009; Clouet et al., 2011; Wang et al., 2015; Li et al., 2017). However, the detailed mechanisms of these processes remain unknown.

LncRNAs are thought to be more than 200 nt long with limited or no protein-coding capacity and are essential modulators in aspects of cell biology through regulation at the posttranscriptional, transcriptional, and chromatin organization levels (Zhang et al., 2018; Zhao et al., 2018; Zou et al., 2018; Yu et al., 2018; Cao et al., 2019; Li et al., 2019). References have noted that lncRNAs play roles in cell molecular functions such as cell differentiation, metastasis, apoptosis, and proliferation (Pan et al., 2018; Xiao et al., 2018; Xu et al., 2018; Yang et al., 2018). The dysregulation of lncRNAs occurs in most types of diseases, including scoliosis, Parkinson’s disease, osteosarcoma, and IDD (Xi et al., 2017; Xu et al., 2018; Boros et al., 2020; Li et al., 2020). Recently, Gao et al. (2019) noted that HOXC13-AS was upregulated in HNSC samples and that HOXC13-AS knockdown suppressed cell invasion, proliferation and invasion by modulating HMGA2/miR-383-3p. Li et al. (2019) noted that HOXC13-AS was overexpressed in breast tumor samples and that HOXC13-AS overexpression induced cell growth by sponging PTEN/miR-497-5p. However, the functional role and mechanism of HOXC13-AS in IDD remain unclear.

We found that HOXC13-AS was overexpressed in IDD specimens compared to control specimens and that HOXC13-AS induced inflammatory cytokine expression and ECM degradation.

## Materials and Methods

### Sample Selection and Cell Transfection

Human IVD specimens from IDD patients and vertebral fracture cases were collected from our hospital. All patients underwent lumbar MRI, and the degree of disc degeneration was analyzed using modified Pfirrmann scoring. The NP cell line was obtained from ScienCell (San Diego, California, United States, No. Catalog #4800) and was cultured in F12/DMEM supplemented with streptomycin, penicillin, and serum. siRNA-NC and ADAMTS5 siRNA, pcDNA-HOXC13-AS and pcDNA-control, miR-497-5p scramble, and the mimics were synthetized by GenePharma. These vectors were transfected into NP cells using Lipofectamine 2000. The study was approved by the ethics committee of Tianjin First Central Hospital and followed the Declaration of Helsinki. Written consents were obtained from all cases.

### Luciferase Assays

miR-497-5p was predicted to link with the ADAMTS5 3′-UTR using TargetScan software. Wild-type (WT) and mutant (Mut)-type 3′ UTR fragments of ADAMTS5 were cloned by PCR. NP cells were cultured in 96-well dishes and cotransfected with Mut ADAMTS5 3′ UTR and WT ADAMTS5 3′ UTR and miR-497-5p scramble and mimic. After 48 h, the luciferase value was analyzed using a luciferase analysis kit (Promega).

### ELISA

The levels of IL-1β, IL-6, and TNF-α in the medium supernatant were analyzed by ELISA (IL-1β, IL-6, and TNF-α, R&D Systems) following the manufacturer’s instructions.

### qRT-PCR

A TRIzol kit (Invitrogen) was used to extract RNA from NP cells and specimens. RT-qPCR analysis was applied to study HOXC13-AS, mRNA, and miR-497-5p expression levels using SYBR Reagent (TaKaRa, Beijing) on an ABI 7300 PCR system (Applied Biosystems, MA). The PCR primers were as follows: HOXC13-AS qF: TCC​CAC​GGC​TTT​CTT​AGG​TCA, HOXC13-AS qR: GAC​TCA​ATT​CCA​CGG​AGA​TGC; ADAMTS5 qF: GAG​GAT​TTA​TGT​GGG​CAT​CAT​TCA​TGT​G, ADAMTS5 qR: CAT​ATG​GTC​CCA​ACG​TCT​GC; miR-497-5p qF: CAG​CAG​CAC​ACT​GTG​GTT​TGT; U6 qF: CTCGCTTCGGCAGCACA, qR: AAC​GCT​TCA​CGA​ATT​TGC​GT; GAPDH qF: GCT​CTC​TGC​TCC​TCC​TGT​TC, qR: ACG​ACC​AAA​TCC​GTT​GAC​TC. U6 was used as a control for miR-497-5p, and GAPDH was applied for other genes.

### Statistical Assay

The results are expressed as the means ± SD. Statistical assays were carried out using SPSS, and significant differences were determined with Student’s *t* test. Spearman’s two-tailed correlation analysis was used for HOXC13-AS and miR-497-5p expression. *p* < 0.05 was set to be statistically significant.

## Results

### TNF-α and IL-1β Induced HOXC13-AS and ADAMTS5 Expression and Suppressed miR-497-5p Expression

**FIGURE 1:**
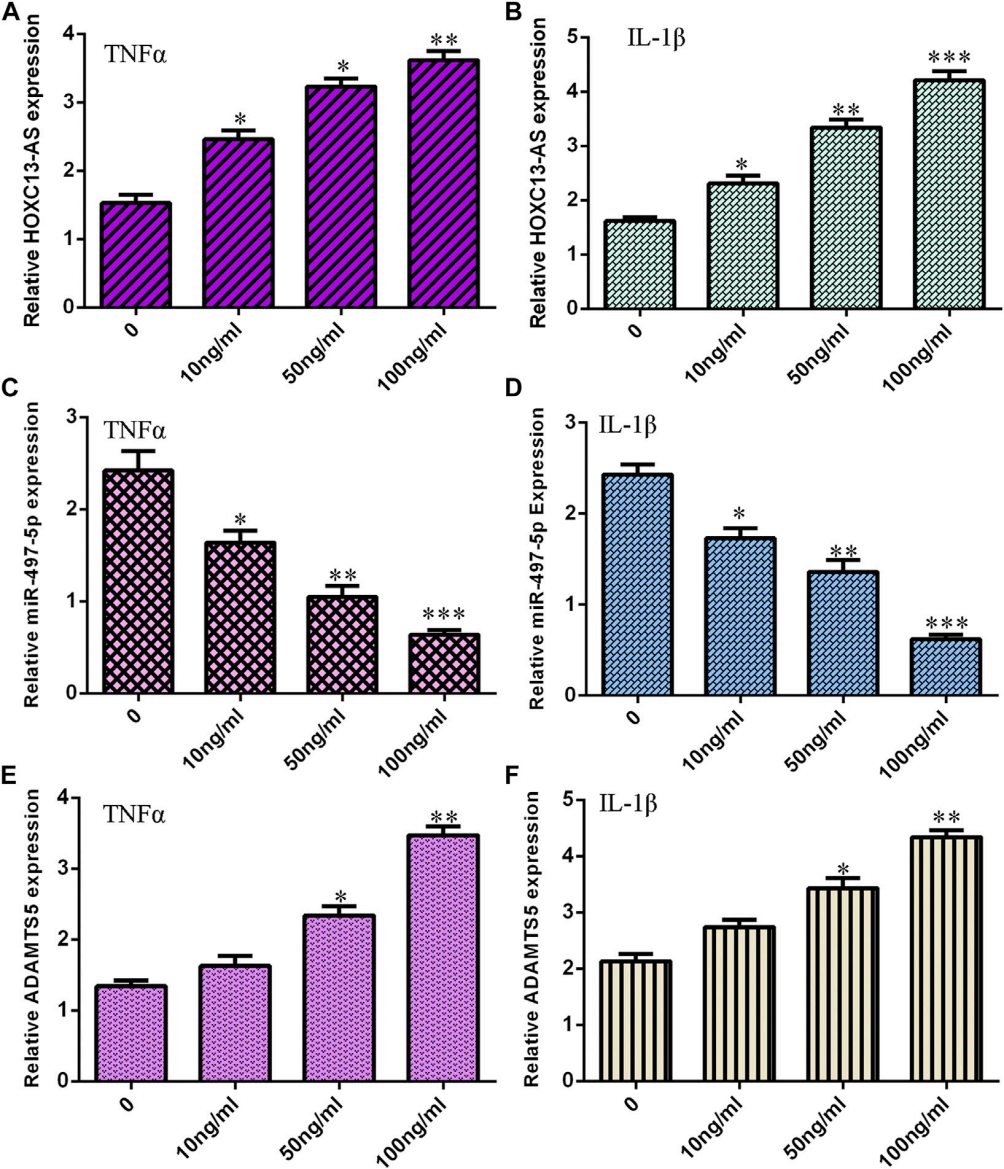
TNF-α and IL-1β induced HOXC13-AS and ADAMTS5 expression and suppressed miR-497-5p expression. **(A)** TNF-α induced HOXC13-AS expression in a dose-dependent manner in NP cells. **(B)** HOXC13-AS expression was measured by qRT-PCR. **(C)** miR-497-5p expression was decreased in NP cells treated with TNF-α. **(D)** miR-497-5p expression was detected by qRT-PCR. **(E)** TNF-α treatment increased ADAMTS5 expression in a dose-dependent manner in NP cells. **(F)** ADAMTS5 expression was determined by qRT-PCR. *p < 0.05, **p < 0.01, and ***p < 0.001.

First, we noted that treatments with TNF-α and IL-1β induced HOXC13-AS expression in a dose-dependent manner in NP cells (Figures 1A,B). The miR-497-5p expression levels were decreased in NP cells treated with TNF-α and IL-1β (Figures 1C,D). Moreover, treatments with TNF-α and IL-1β increased ADAMTS5 expression in a dose-dependent manner in NP cells (Figures 1E,F).

### HOXC13-AS was Upregulated in IDD Specimens

**FIGURE 2:**
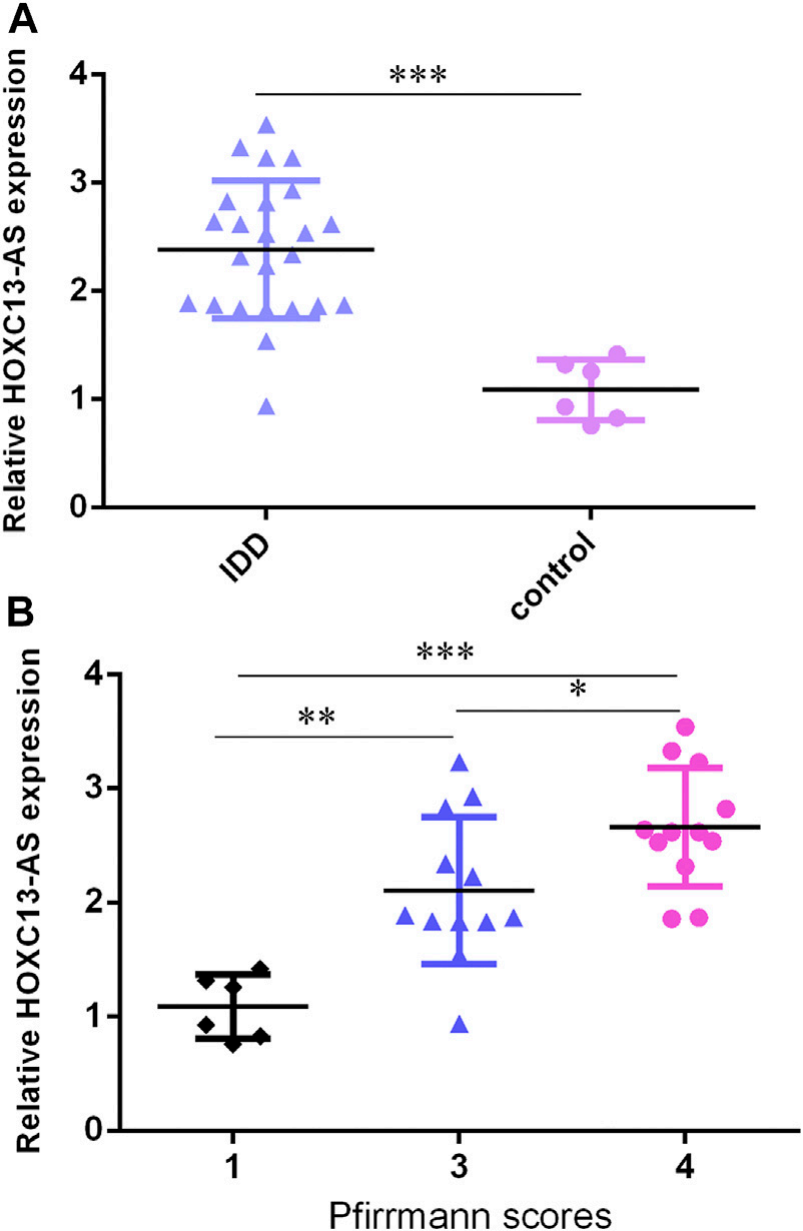
HOXC13-AS was upregulated in IDD specimens. **(A)** HOXC13-AS was overexpressed in IDD specimens compared to control specimens by RT-qPCR. **(B)** Higher expression of HOXC13-AS was correlated with high Pfirrmann scores. *p < 0.05, **p < 0.01, and ***p < 0.001.

We then determined that HOXC13-AS was overexpressed in IDD specimens compared to control specimens by RT-qPCR (Figure 2A). Moreover, the higher expression of HOXC13-AS was correlated with high Pfirrmann scores (Figure 2B).

### miR-497-5p was Downregulated in IDD Specimens

**FIGURE 3:**
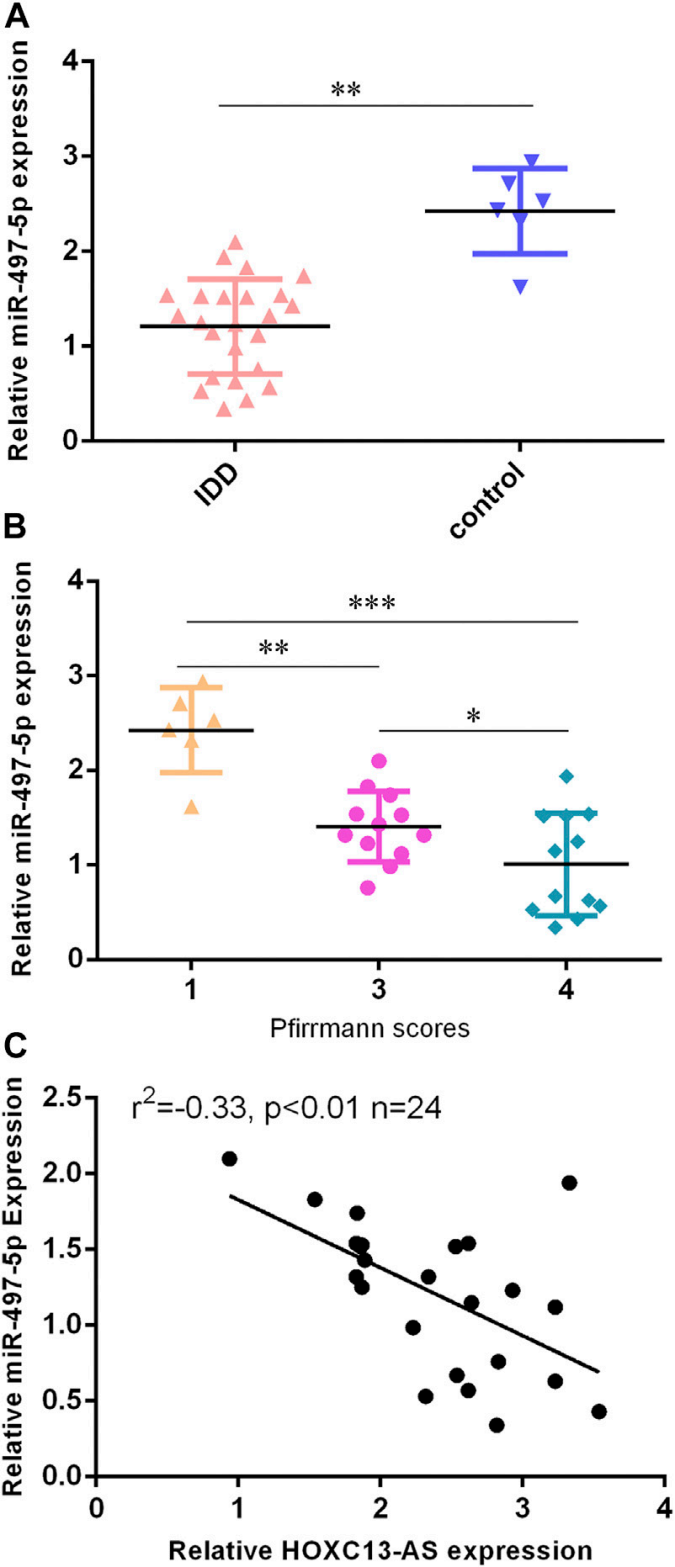
miR-497-5p was downregulated in IDD specimens. **(A)** miR-497-5p was downregulated in IDD specimens compared to control specimens by RT-qPCR. **(B)** Lower expression of miR-497-5p was correlated with high Pfirrmann scores. **(C)** miR-497-5p expression was negatively proportional to HOXC13-AS expression in IDD specimens. *p < 0.05, **p < 0.01, and ***p < 0.001.

Then, we found that miR-497-5p was downregulated in IDD specimens compared to control specimens by RT-qPCR (Figure 3A). Moreover, the lower expression of miR-497-5p was correlated with high Pfirrmann scores (Figure 3B). The miR-497-5p level was negatively proportional to HOXC13-AS expression in IDD specimens (Figure 3C).

### miR-497-5p Targets ADAMTS5 Expression in NP Cells

**FIGURE 4:**
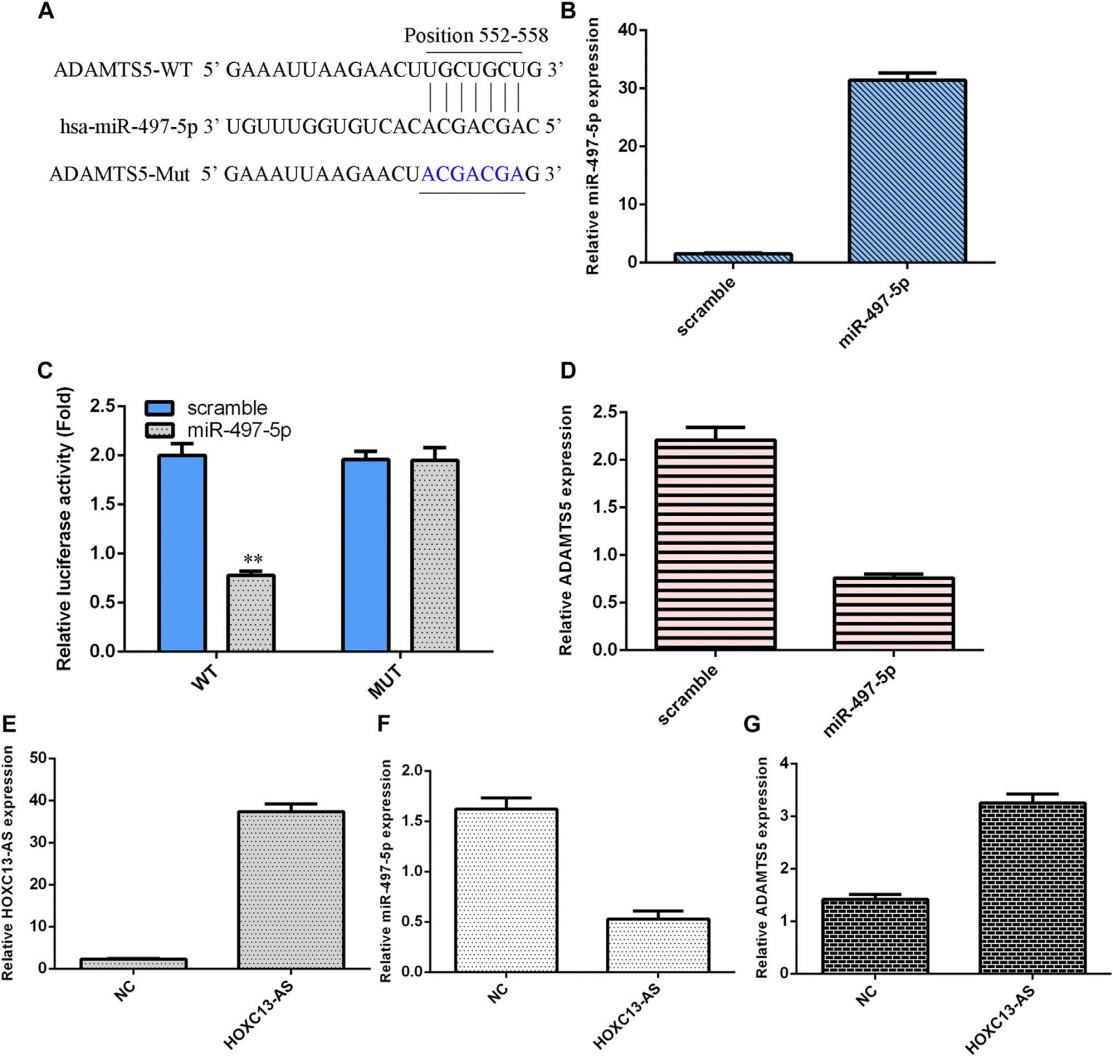
miR-497-5p targets ADAMTS5 expression in NP cells. **(A)** miR-497-5p was predicted to link with the ADAMTS5 3′-UTR using TargetScan software. **(B)** miR-497-5p was obviously upregulated in NP cells after treatment with the miR-497-5p mimic. **(C)** Luciferase analysis data showed that miR-497-5p overexpression inhibited the luciferase expression of the wild-type reporter gene but not the mutated 3′UTR vector. **(D)** Ectopic miR-497-5p expression decreased ADAMTS5 levels in NP cells. **(E)** HOXC13-AS expression was measured by qRT-PCR. **(F)** Upregulation of HOXC13-AS expression inhibited miR-497-5p expression in NP cells. **(G)** ADAMTS5 expression was measured by qRT-PCR. **p < 0.01.

We utilized TargetScan software to predict that miR-497-5p was linked to the ADAMTS5 3′-UTR (Figure 4A). miR-497-5p was obviously upregulated in NP cells after treatment with the miR-497-5p mimic (Figure 4B). Luciferase analysis data suggested that miR-497-5p overexpression inhibited the luciferase value of the wild-type reporter gene but not the mutated 3′UTR vector (Figure 4C). Ectopic miR-497-5p expression decreased ADAMTS5 levels in NP cells (Figure 4D). HOXC13-AS was obviously upregulated in NP cells after treatment with the pcDNA-HOXC13-AS vector (Figure 4E). Upregulation of HOXC13-AS expression inhibited miR-497-5p expression in NP cells (Figure 4F). Overexpression of HOXC13-AS suppressed ADAMTS5 expression in NP cells (Figure 4G).

### HOXC13-AS Induced Inflammatory Cytokine Expression and ECM Degeneration

**FIGURE 5:**
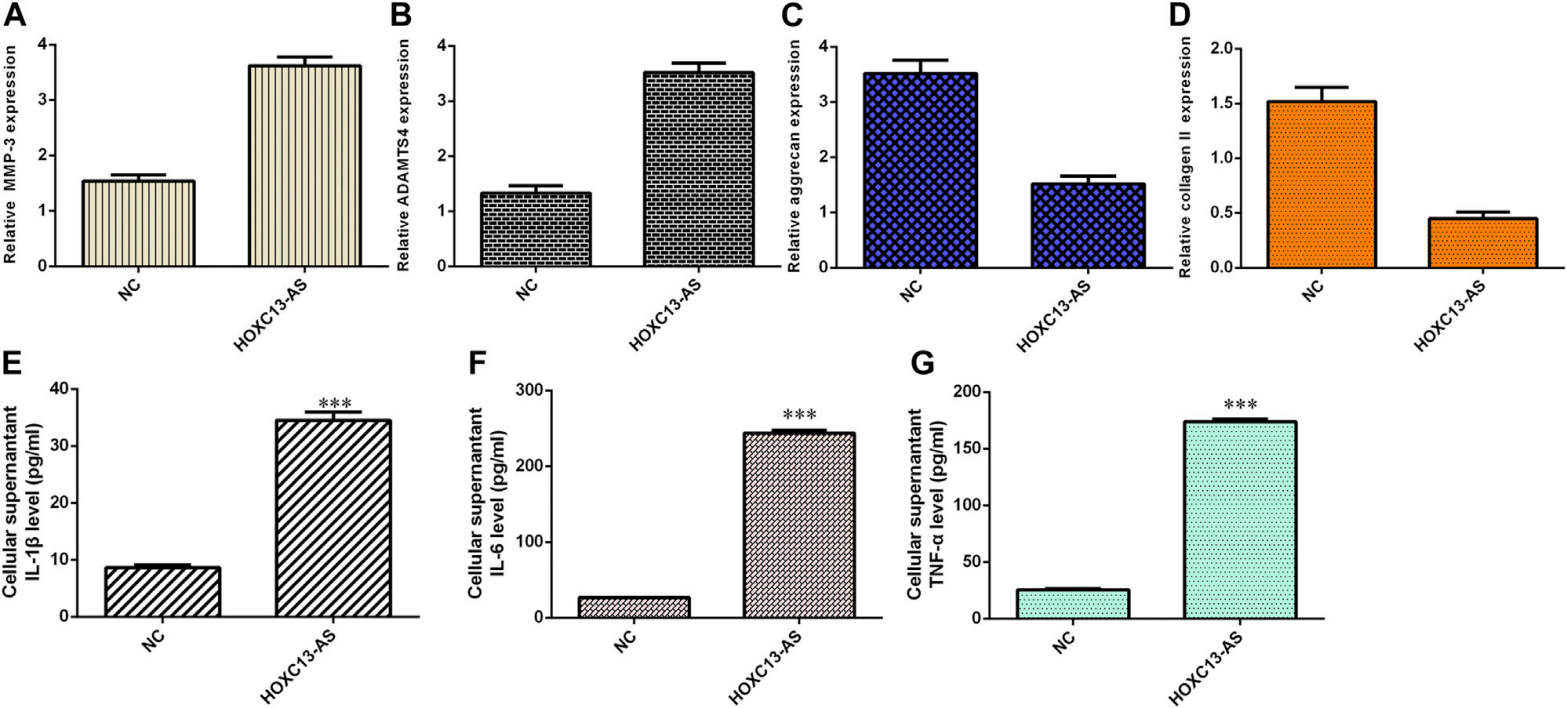
HOXC13-AS induced inflammatory cytokine expression and ECM degeneration. **(A)** MMP-3 expression was measured by qRT-PCR. **(B)** Ectopic expression of HOXC13-AS promoted ADAMTS4 expression in NP cells. **(C)** Aggrecan expression was determined by qRT-PCR. **(D)** Collagen II expression was measured by qRT-PCR. **(E)** IL-1β expression was determined by ELISA. **(F)** IL-6 expression was determined by ELISA. **(G)** TNF-α expression was determined by ELISA. ***p < 0.001.

Ectopic expression of HOXC13-AS enhanced MMP-3 (Figure 5A) and ADAMTS4 (Figure 5B) expression in NP cells. Overexpression of HOXC13-AS decreased aggrecan (Figure 5C) and collagen II (Figure 5D) expression in NP cells. By ELISA, we determined that elevated expression of HOXC13-AS increased the expression levels of three inflammatory cytokines, IL-1β (Figure 5E), IL-6 (Figure 5F), and TNF-α (Figure 5G).

### miR-497-5p Suppressed Inflammatory Cytokine Expression and ECM Degeneration

**FIGURE 6:**
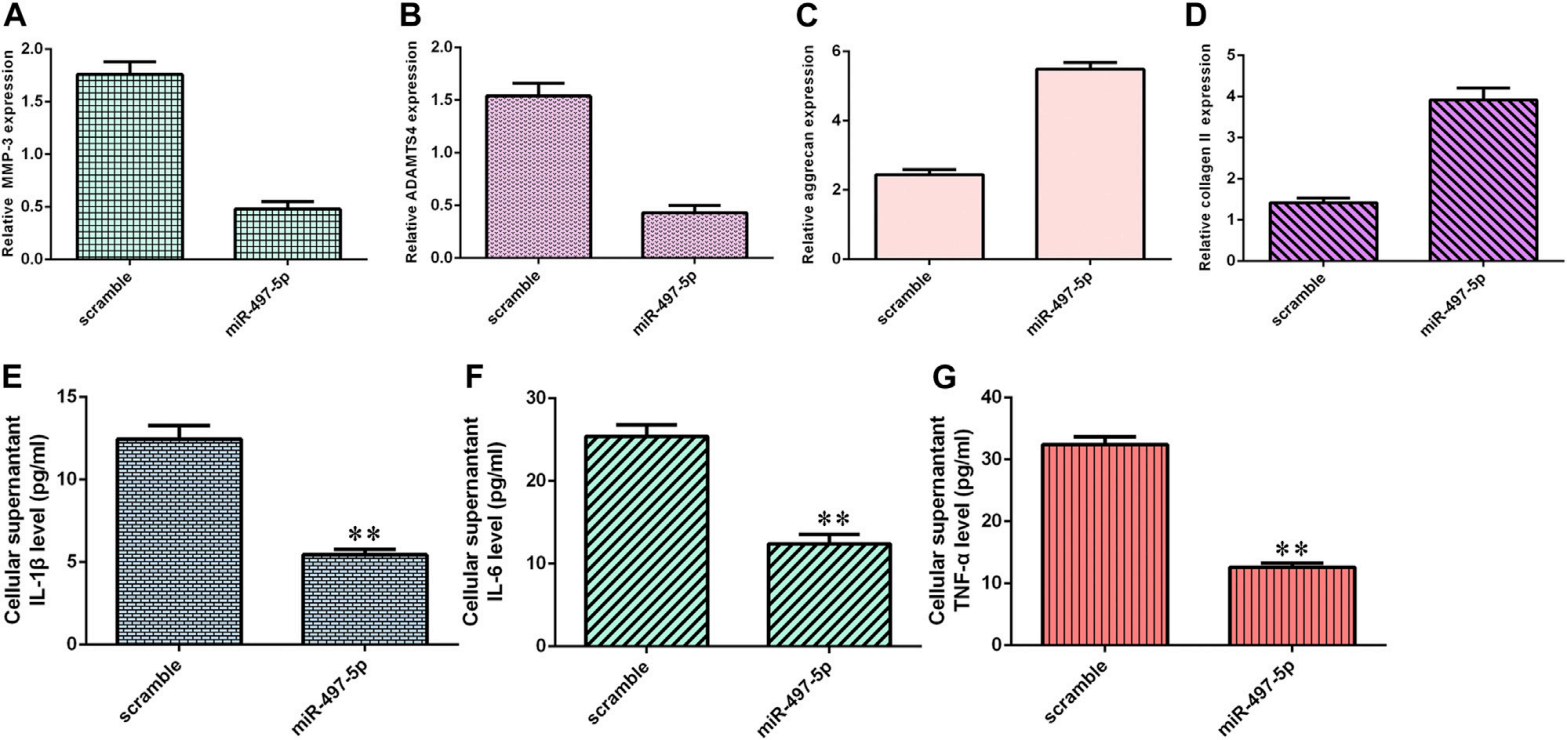
miR-497-5p suppressed inflammatory cytokine expression and ECM degeneration. **(A)** MMP-3 expression was detected by qRT-PCR. **(B)** Overexpression of miR-497-5p suppressed ADAMTS4 expression in NP cells. **(C)** Aggrecan expression was measured by qRT-PCR. **(D)** Collagen II expression was detected by qRT-PCR. **(E)** IL-1β expression was determined by ELISA. **(F)** IL-6 expression was determined by ELISA. **(G)** TNF-α expression was determined by ELISA. **p < 0.01.

Overexpression of miR-497-5p inhibited MMP-3 (Figure 6A) and ADAMTS4 (Figure 6B) expression in NP cells. Ectopic expression of miR-497-5p enhanced aggrecan (Figure 6C) and collagen II (Figure 6D) expression in NP cells. By ELISA, we showed that elevated expression of miR-497-5p increased the expression levels of three inflammatory cytokines, IL-1β (Figure 6E), IL-6 (Figure 6F), and TNF-α (Figure 6G).

### HOXC13-AS Induced Inflammatory Cytokine Expression and ECM Degradation by Modulating miR-497-5p/ADAMTS5

**FIGURE 7:**
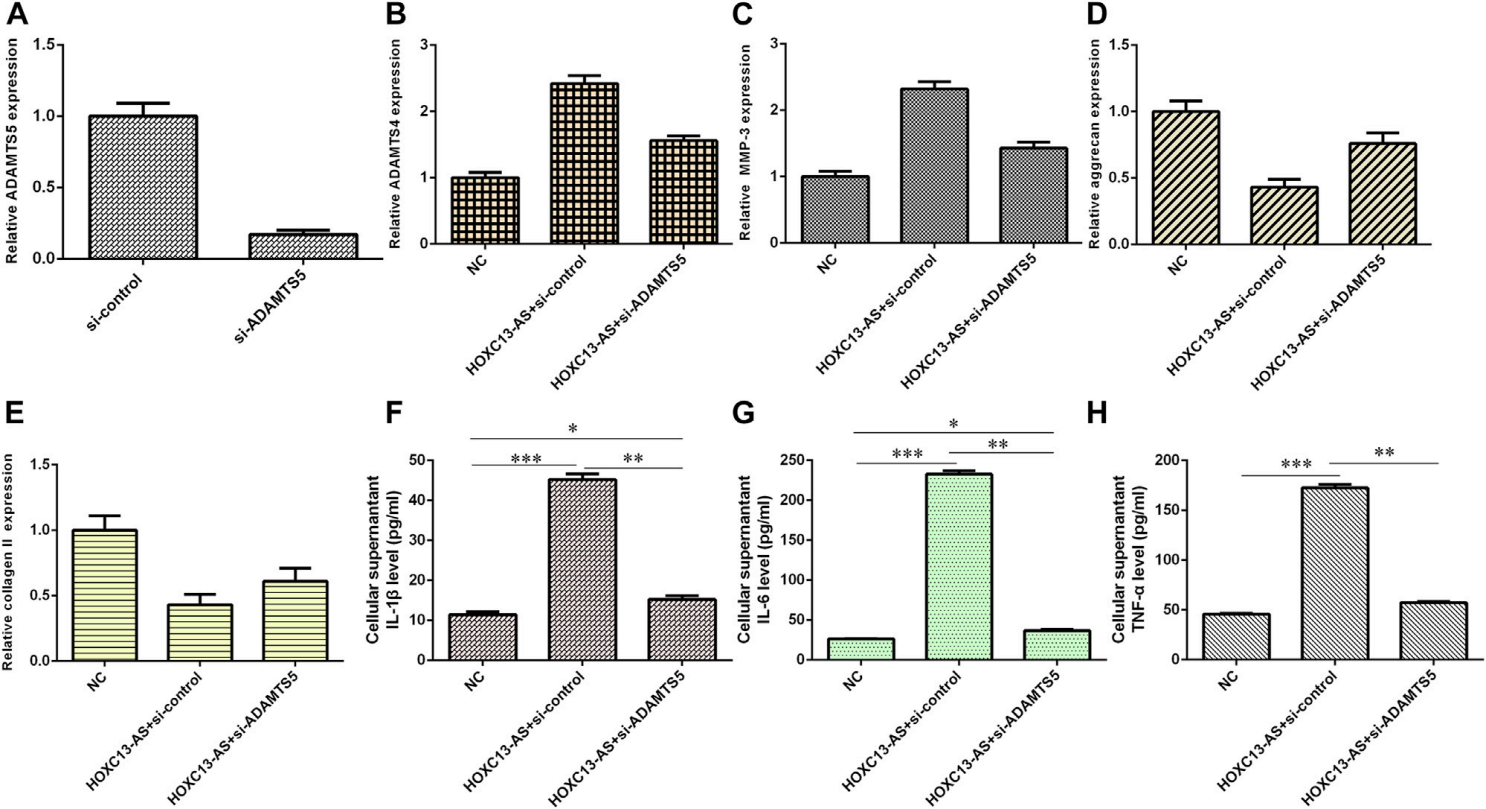
HOXC13-AS induced inflammatory cytokine expression and ECM degradation by modulating miR-497-5p/ADAMTS5. **(A)** ADAMTS5 was obviously downregulated in NP cells after treatment with ADAMTS5 siRNA. **(B)** ADAMTS4 expression was measured by qRT-PCR. **(C)** MMP-3 expression was measured by qRT-PCR. **(D)** Aggrecan expression was measured by qRT-PCR. **(E)** OXC13-AS overexpression inhibited collagen II expression, while downregulation of ADAMTS5 expression enhanced this effect. **(F)** IL-1β expression was determined by ELISA. **(G)** IL-6 expression was determined by ELISA. **(H)** TNF-α expression was determined by ELISA. *p < 0.05, **p < 0.01, and ***p < 0.001.

We then explored the effects of three different treatment conditions on inflammatory cytokine expression and ECM degradation in NP cells. ADAMTS5 was obviously downregulated in NP cells after treatment with ADAMTS5 siRNA (Figure 7A). HOXC13-AS promoted ADAMTS4 and MMP-3 expression, while ADAMTS5 siRNA inhibited this function (Figures 7B,C). HOXC13-AS overexpression inhibited aggrecan and collagen II expression, while downregulation of ADAMTS5 expression enhanced this effect (Figures 7D,E). Elevated expression of HOXC13-AS promoted the expression of three inflammatory cytokines, IL-1β, IL-6, and TNF-α, while inhibition of ADAMTS5 expression decreased this effect (Figures 7F–H).

## Discussion

Recently, abundant references have illustrated that lncRNA dysregulation is involved in the development of several diseases, including IDD (Li et al., 2018). In Ruan’s study, p53, p21, and NEAT1 were overexpressed in IDD samples, and ectopic expression of NEAT1 promoted ECM degradation by regulating MAPK/ERK1/2 pathway signaling expression (Ruan et al., 2018). Wang showed that linc-ADAMTS5 was negatively correlated with RREB1 to suppress ADAMTS5 and ECM degeneration in IDD (Wang K. et al., 2017). Moreover, Wang X. B. et al. (2017) found that RP11-296A18.3 was overexpressed in IDD samples and that RP11-296A18.3 knockdown decreased NP cell growth and ECM synthesis by modulating miR-138/HIF1A expression. Gao et al. (2019) noted that HOXC13-AS was upregulated in HNSC samples and that HOXC13-AS knockdown suppressed cell invasion, proliferation, and invasion by modulating HMGA2/miR-383-3p. Li X. W. et al. (2019) noted that HOXC13-AS was overexpressed in breast tumor samples and that HOXC13-AS overexpression induced cell growth by sponging PTEN/miR-497-5p. Our research revealed that TNF-α and IL-1β induced HOXC13-AS expression in NP cells. HOXC13-AS was overexpressed in IDD specimens compared to control specimens, and higher expression of HOXC13-AS was correlated with high Pfirrmann scores. Ectopic expression of HOXC13-AS promoted MMP-3 and ADAMTS4 and inhibited aggrecan and collagen II expression in NP cells. Overexpression of miR-497-5p suppressed inflammatory cytokine expression and ECM degeneration in NP cells. Furthermore, overexpression of HOXC13-AS increased the expression levels of three inflammatory cytokines, IL-1β, IL-6, and TNF-α.

LncRNAs act as posttranscriptional modulators of miRNA expression by functioning as “sponges” (Bian et al., 2017; Tian et al., 2018; Zhang et al., 2018; Cao et al., 2019). In line with previous data, we noted that upregulation of HOXC13-AS expression inhibited miR-497-5p expression in NP cells (Li et al., 2019). Furthermore, we utilized TargetScan software to predict that miR-497-5p was linked to the ADAMTS5 3′-UTR. Luciferase analysis data suggested that ADAMTS5 was a direct gene of miR-497-5p. Previous studies have suggested that ADAMTS5 plays critical roles in the progression of IDD (Ngo et al., 2017; Wang et al., 2018). In Seki’s study, they showed that ADAMTS5 siRNA injection inhibited NP sample degradation and ameliorated histologic and MRI grades (Seki et al., 2009). Zhao et al. (2011) indicated that IL-1β promoted ADAMTS-5 expression in NP cells. Our results illustrated that TNF-α and IL-1β induced ADAMTS5 expression and suppressed miR-497-5p expression. miR-497-5p was downregulated in IDD specimens compared to control specimens, and the lower expression of miR-497-5p was correlated with high Pfirrmann scores. The miR-497-5p expression level was negatively proportional to HOXC13-AS expression in IDD specimens. HOXC13-AS induced inflammatory cytokine expression and ECM degradation by modulating miR-497-5p/ADAMTS5.

To conclude, we found that HOXC13-AS was overexpressed in IDD specimens compared to control specimens and that HOXC13-AS induced inflammatory cytokine expression and ECM degradation by modulating miR-497-5p/ADAMTS5. These results suggest that HOXC13-AS may be a treatment target for IDD.

## Data Availability

The raw data supporting the conclusions of this article will be made available by the authors, without undue reservation.
